# Anti-CD123 antibody-modified niosomes for targeted delivery of daunorubicin against acute myeloid leukemia

**DOI:** 10.1080/10717544.2017.1333170

**Published:** 2017-06-02

**Authors:** Fu-rong Liu, Hui Jin, Yin Wang, Chen Chen, Ming Li, Sheng-jun Mao, Qiantao Wang, Hui Li

**Affiliations:** a Key Laboratory of Drug Targeting and Drug Delivery System, Ministry of Education and West China School of Pharmacy, Sichuan University, Chengdu, China;; b Department of Hematology, Sichuan Academy of Medical Sciences and Sichuan Provincial People Hospital, Chengdu, China

**Keywords:** Niosome, CD123, drug targeting, acute myeloid leukemia, daunorubicin

## Abstract

A novel niosomal delivery system was designed and investigated for the targeted delivery of daunorubicin (DNR) against acute myeloid leukemia (AML). Anti-CD123 antibodies conjugated to Mal-PEG_2000_-DSPE were incorporated into normal niosomes (NS) via a post insertion method to afford antibody-modified niosomes (CD123-NS). Next, NS was modified with varying densities of antibody (0.5 or 2%, antibody/Span 80, molar ratio), thus providing L-CD123-NS and H-CD123-NS. We studied the effect of antibody density on the uptake efficiency of niosomes in NB4 and THP-1 cells, on which CD123 express differently. Our results demonstrate CD123-NS showed significantly higher uptake efficiency than NS in AML cells, and the uptake efficiency of CD123-NS has been ligand density-dependent. Also, AML cells preincubated with anti-CD123 antibody showed significantly reduced cellular uptake of CD123-NS compared to control. Further study on the uptake mechanism confirmed a receptor-mediated endocytic process. Daunorubicin (DNR)-loaded H-CD123-NS demonstrated a 2.45- and 3.22-fold higher cytotoxicity, compared to DNR-loaded NS in NB4 and THP-1 cells, respectively. Prolonged survival time were observed in leukemic mice treated with DNR-H-CD123-NS. Collectively, these findings support that the CD123-NS represent a promising delivery system for the treatment of AML.

## Introduction

Acute myeloid leukemia (AML), a heterogeneous clonal disorder of hemopoietic progenitor cells (Estey & Döhner, 2006), has been one of the most common myeloid leukemia in adults with over 13,000 individuals diagnosed each year in the United States (Society, 2013; Cheng et al., 2014). According to American Cancer Society statistics, 19,950 new cases of AML were reported in 2016, which accounted for 33% of all leukemia and 75% of all acute leukemia, and 10,430 people would die of the disease (Siegel et al., 2016). Standard induction treatment for AML has been chemotherapy with combinations of anthracycline, such as daunorubicin (DNR) or idarubicin and cytarabine since 1970 s (Bob Löwenberg et al., 2003; Stone et al., 2004; Tallman et al., 2005). However, only 26.6% of AML patients survived for over 5 years from 2006 to 2012 (Institute, 2016), and patients over age 60 have a poor prognosis with 10% or less 5-year survival (Tallman & Stein, 2012) mainly due to the persistence or relapse of AML.

Recently, increasing evidence supported that leukemic stem cells (LSCs) account for the high rate of therapeutic failure (Tettamanti et al., 2014). LSCs are the only AML cells that are capable of self-renewal while generating rapidly proliferating progenitors and terminal leukemic blasts (Jin et al., 2009). LSCs mostly remain in G0 phase of the cell cycle, and are probably the reason for low rates of long-term remission, high relapse and multidrug resistance for AML (Misaghian et al., 2009; Becker & Jordan, 2011; Konopleva & Jordan, 2011; Zhou & Chng, 2014). Hence, the development of new therapies that selectively target AML cells and LSCs, while sparing the normal counterpart of hematopoietic stem/progenitor cells (HSPCs), is of great significance for AML treatment.

CD123 is the α subunit of interleukin-3 receptor (IL3R), which is expressed across AML blasts, CD34^+^ leukemic progenitors, and AML-LSCs but hardly on normal HSCs (Jordan et al., 2000; Florian et al., 2006; He et al., 2015), thus rendering CD123 a potential target for AML cells. Recent studies have also reported that 77.9% (232/298) AML samples were positive for CD123 (Ehninger et al., 2014). Moreover, the over-expression of CD123 on AML cells was associated with resistance to apoptosis, higher proliferating potential and poor prognosis (Testa et al., 2002; Vergez et al., 2011). In a previous study, CD123-directed monoclonal antibodies (7G3) have been shown the potential to target AML LSCs in NOD/SCID mice, reducing AML stem cells engraftment and improving survival (Jin et al., 2009). These findings suggest that CD123 could be a promising cell-surface target for therapeutic intervention of AML.

Recently, greater efforts have been made to CD123 monoclonal antibody-based therapies against AML, e.g., the antibody-drug conjugates (ADCs). ADCs consist of highly specific antibodies and potent small molecule drugs, which are covalently conjugated via lysine or cysteine residues (Gebleux & Casi, 2016). Anti-CD123 antibody drug conjugates (CD123-CPT) were developed by integrating anti-CD123 antibody with camptothecin (CPT) via a disulfide linker (Li et al., 2016). Despite the demonstrated efficacy, such application suffered from a series of limitations (Gebleux & Casi, 2016). The instability of the linker has negative impact on ADC efficacy and therapeutic window, which often leads to serious ‘off-target’ toxicities and even failure in clinical trials (Tsuchikama & An, 2016). Mylotarg® was withdrawn from the market in 2010 due to a lack of clinical benefit and high fatal toxicity rate compared to the standard chemotherapy (ten Cate et al., 2009). Thus, seeking alternative therapeutic option with improved efficacy and reduced off-target toxicity remains a great challenge for AML treatment.

Niosomes are vesicles composed of nonionic surfactants, which are biodegradable, relatively nontoxic, stable and inexpensive (Hasan et al., 2013). As an alternative to liposomes, they are capable to accommodate drug molecules with a wide range of solubility due to the presence of hydrophilic, amphiphilic and lipophilic moieties in their structure (Kazi et al., 2010; Abdelkader et al., 2014). Given these advantages, there is increasing interest in niosomes as drug carriers (Hong et al., 2009; Manjappa et al., 2011; Tavano et al., 2014; Sun et al., 2015).

In this study, an anti-CD123 antibody conjugated niosomal formulation (CD123-NS) was designed and fabricated. Furthermore, the anti-tumor activity and cellular uptake of CD123-NS versus normal niosome were assessed using AML cells and Leukemic mice over-expressing CD123. We report here on the preparation and characterization of CD123-NS, as well as its AML-targeting properties.

## Materials and methods

### Materials and animals

The nonionic surfactant sorbitan monoleate, Span 80 was a Solarbio product (Beijing, China). Cholesterol (Chol) and 1,2-distearoyl-sn-glycero-3-phosphoethanolamine-*N*-[maleimide(polyethylene glycol)-2000] (Mal-PEG_2000_-DSPE) were obtained from Avanti Polar Lipids (Alabaster, AL). Daunorubicin hydrochloride (DNR-HCl) was purchased from Shanghai Baili Biotechnology Co. Ltd. (Shanghai, China) with greater than 97% purity. Coumarin-6, 2-iminothiolane (Traut’s reagent), [3-(4, 5-dimethylthiazol-2-yl)-2, 5-diphenyl] tetrazolium bromide (MTT), 4, 6-diamidino-2-phenylindole (DAPI) and dialysis bag (MWCO, 7000) were purchased from Sigma-Aldrich Chemical Co. (St. Louis, MO). Sephadex G-50 was purchased from Amersham Pharmacia Biotech (Stockholm, Sweden) and Sepharose CL-4B from Yuanye Biotech (Shanghai, China). Purified mouse anti-human CD123 monoclonal antibody (mAb) (clone 7G3), mouse anti-human CD123-APC and mouse IgG2a-APC were purchased from Becton Dickinson (New Jersey). The secondary antibodies, anti-mouse IgG (H + L) Alexa fluor 647 were obtained from Cell Signaling Technology (Beverly, MA). Bicinchoninic acid (BCA) protein assay kit was procured from KeyGEN Biotech (Nanjing, China). All other chemicals and reagents used were of analytical grade or better and were obtained commercially.

Six to seven-week-old male non-obese diabetic/severe combined immunodeficient (NOD/SCID) mice (weighing 20–25 g) were purchased from the Experimental Animal Center of Sichuan University (China) and housed in a standard pathogen-free (SPF) conditions for a week prior to experiments. All animal studies were performed in accordance with the principles of care and use of laboratory animals and were approved by the Experimental Animal Administrative Committee of Sichuan University.

### Cell lines

The human acute promyelocytic leukemia cell line, NB4 (FAB-M3) and the acute monocytic leukemia cell line, THP-1 (FAB-M5) were purchased from the American Type Culture Collection (ATCC, Manassas, VA). Both NB4 and THP-1 cells were maintained in Roswell Park Memorial Institute (RPMI) media (Gibco Invitrogen Carlsbad, CA) supplemented with 10% fetal bovine serum (FBS), 100 units/ml penicillin and 100 mg/ml streptomycin at 37 °C under a humidified atmosphere of 95% air and 5% CO_2_.

### Vehicle preparation

#### Preparation of niosomes(NS)

Prior to drug encapsulation, the commercially obtained DNR-HCl was desalted as previously reported (Altreuter et al., 2002). Niosomes were prepared following a thin-film hydration method (Tavano et al., 2013; Yeom et al., 2014). Accurately weighed Span 80 (0.03 mmol) and cholesterol (0.01 mmol) were dissolved in chloroform in a round-bottom flask and vacuum evaporated at 40 °C. The resulting dried film was then hydrated with 4 ml of 0.01 M pH 7.4 PBS for 30 min at 40 °C and subjected to probe sonication to obtain blank vesicles. DNR-/Coumarin-6-NS were prepared similarly using chloroform containing 1.2 mg/ml DNR or 0.04 mg/ml Coumarin-6. The niosomes encapsulated with DNR or Coumarin-6 were separated from free DNR or Coumarin-6 using a Sephadex G-50 column and stored in dark atmosphere at 4 °C for later use.

#### Preparation of anti-CD123 antibody-conjugated niosomes (CD123-NS)

CD123-NS were prepared using a post-insertion method as previously described (Yang et al., 2007; Al-Ahmady et al., 2014). Anti-CD123 antibody was first thiolated with Traut's reagent at a molar ratio of 1:100 for 1 h under continuous stirring at room temperature in pH8.0 deoxygenated HEPES. Unreacted Traut’s reagent was removed through dialysis against deoxygenated HEPES (pH 7.4) for 4 h. Mal-PEG_2000_-DSPE was dissolved in chloroform synchronously and vacuum evaporated to form a thin lipid film. The coupling reaction was performed by adding the obtained thiolated Ab solutions to Mal-PEG_2000_-DSPE film at 1:10 molar ratio (Ab:Mal-PEG_2000_-DSPE) and incubating overnight at room temperature. Anti-CD123-Mal-PEG_2000_-DSPE micelles were then post-inserted into preformed vesicles at two different antibody/Span 80 molar ratios (0.5 and 2%) by incubating overnight at room temperature to afford L-CD123-NS (low density of anti-CD123 Ab modification) and H-CD123-NS (high density of anti-CD123 Ab modification). The CD123-NS were separated from the unconjugated anti-CD123 antibody and antibody-conjugated Mal micelles using Sepharose CL-4B columns in pH 7.4 HEPES. The amount of antibody post-inserted into vesicles was then determined by BCA protein assay according to the manufacturer’s instructions. All above reactions were performed at oxygen free conditions.

### Niosomes characterization

#### Size and zeta potential measurements

The mean size and zeta potential of the particles were measured by dynamic laser scattering (DLS) using a Malvern Zetasizer Nano ZS90 (Malvern Instruments, Worcestershire, UK). Prior to detection, each sample was diluted by 10-fold using the same buffer solution.

#### Drug encapsulation efficiency (EE%)

The amount of DNR encapsulated in the vesicles was measured using high performance liquid chromatography (HPLC) (Agilent 1100) with a C18 reverse phase column (Diamonsil C18, 150 × 4.6 mm, Dikma Technologies, IL) at a detection wavelength of 481 nm. Briefly, following elution to remove the free drug, the equal purified and untreated samples were spin-dried and dissolved in 1 ml of chloroform. The solution then was filtered using 0.22 μm syringe filter prior to HPLC analysis. The concentration of Coumarin-6 was quantified using fluorescence spectroscopy at 466_Ex_/504_Em_ (RF-5301 spectrofluorometer, Shimadzu, Japan) likewise. Encapsulation efficiency (% EE) was calculated by comparing the total response value (peak area or fluorescence intensity) of DNR/Coumarin-6 pre- and post gel filtration, diluted to the same final lipid concentration.

### *In vitro* release study

After separation of the free DNR, each niosome preparation was transferred to a dialysis bag (MWCO, 7000) immersed in 100 ml PBS (0.1 M, pH 7.4) containing 0.1% w/v Tween-80 and magnetically stirred at 120 rpm in a water bath at 37 °C. At given time intervals, 2 ml of samples withdrawn from the dialysis medium were replaced by an equal volume of fresh PBS. The drug content was determined by HPLC as described above.

### Analyzing CD123 cellular levels

To quantify the cell surface levels of CD123, 1 × 10^6^ cells of each sample were stained with 2 μl mouse anti-human CD123-APC or IgG2a-APC isotype control antibody for 30 min at 4 °C away from light. The IgG2a isotype control antibodies were used to establish gating parameters for positive cells. Cells were then washed twice with cold PBS and analyzed by flow cytometry (BD FACSCalibur, San Jose, CA). Data was analyzed using FlowJo 7.6.1 cytometry analysis software. (Tree Star, Ashland, OR).

### *In vitro* cellular uptake study

#### Flow cytometry analysis

For a quantitative evaluation of cellular uptake. NB4 and THP-1 cells were seeded into 24-well plates at a density of 1 × 10^6^ cells per well in the absence of FBS and incubated with 35 μl Coumarin-6 loaded vesicles (NS, L-CD123-NS and H-CD123-NS) at a final Coumarin-6 concentration of 40 ng/ml (the group of L-CD123-NS) or 10 ng/ml (the group of H-CD123-NS) for 4 h at 37 °C or 4 °C, respectively. For competitive binding studies, 5 μl (500 μg/500 μl) free anti-CD123 or IgG2a isotype control antibody were added to each well 1 h prior to the vesicles administration. After 4 h incubation, cells were collected and washed twice with cold PBS, and directly used for flow cytometry analysis (BD FACSCalibur, San Jose, CA).

#### Confocal microscopy analysis

For a qualitative evaluation of cellular uptake, the washed cells were transferred to slides coated with poly L-lysine (Hebei Bio-high Technology, China), and fixed with 4% paraformaldehyde solution for 30 min at room temperature, permeabilised with 0.5% triton X-100 for 10 min and incubated with 3% BSA for 30 min to block nonspecific proteins. The cells were then washed twice with cold PBS and incubated with a second antibody for 30 min protected from light. The nuclei were stained with DAPI for 10 min. The monolayer cell was washed twice with cold PBS and analyzed by confocal laser scanning microscopy (CLSM, FV1000, Olympus, San Diego, CA).

### *In vitro* cytoxicity study

The cytotoxicity of DNR loaded vesicles (NS, L-CD123-NS and H-CD123-NS) and free DNR to NB4 and THP-1 cells were assayed using a MTT test according to the manufacturer’s instructions. Briefly, 100 μl cells (1 × 10^6^ cells/ml) in the logarithmic growth phase were incubated with each of the DNR-loaded vesicles and free drug. Cytotoxicity was assessed at 0.375, 0.75, 1.5, 3, 6 and 12 μg/ml DNR after 24 h incubation. At the end of incubation, 20 μl MTT solution (5 mg/ml in pH 7.4 PBS) was added to each well and cells were incubated at 37 °C for another 4 h. Finally, 100 μl of formazan-dissolving buffer (10% sodium dodecyl sulfonate, 5% isobutanol and 0.01 M hydrochloric acid) was added to each well and the absorbance was measured at a wavelength of 570 nm with microplate reader (Bio-Rad, Richmond, CA).

### *In vivo* survival experiment

As previously reported (Li et al., 2014), the AML tumor model was established by injecting 1 × 10^7^ THP-1 cells in the lateral tail vein of the irradiated NOD/SCID mice. After verifying the proliferation of AML cells by flow cytometry (Rombouts et al., 2000), the mice were randomly divided into four equal groups with eight mice in each group. One week later, the animals were administered with free DNR, DNR-NS, or DNR-H-CD123-NS (i.v., 3 mg DNR/kg body weight) in 200 μl saline or saline alone twice a week. The DNR concentration of free DNR, DNR-NS, or DNR-H-CD123-NS saline solution were range from 0.3 to 0.375 mg/ml to fit the body weight of NOD/SCID mice. The animals were monitored and were euthanized when they developed hind-leg paralysis. Survival time was recorded and analyzed by Graph Pad Prism software.

### Statistical analysis

All experiments were performed in triplicates, and the results are expressed as the mean ± SD unless otherwise indicated. Statistical analysis of the data was performed using GraphPad Prism software (v5.0). Statistical comparisons were performed by one-way ANOVA for multiple groups. A *p* value of < .01 and < .05 were considered indications of statistically significant and statistical difference, respectively.

## Results and discussion

### Preparation and characterization of the vesicles

The linkage of anti-CD123 antibody to niosomes is analogous to the coupling on the distal end of PEG groups to immunoliposomes (Yang et al., 2007). The thiolated anti-CD123 antibody was conjugated with the Mal-PEG_2000_-DSPE via thiol-ether bond. A post-insertion method was applied to incorporate the antibody-lipid conjugate onto the niosomal surface via the hydrophobic DSPE domains. BCA assays showed that ∼0.45 and 1.63% of antibody content (%Ab/Span 80) was immobilized onto L-CD123-NS and H-CD123-NS, respectively. DLS measurement showed that the modified niosomes (L-CD123-NS and H-CD123-NS) had a mean particle size about 139.8 ± 4.6 and 146.7 ± 5.3 nm, respectively, similar to that of the normal niosomes(142. 2 ± 3.7 nm), indicating that the coupling process did not greatly affect the size of the vesicles. All obtained NS with or without antibody conjugation showed PDI values ranging from 0.170 to 0.196, which suggested a relatively narrow distribution. The zeta potential of the vesicles was approximately −58 mv, indicating that these particles could remain stable for *in vitro* storage due to electrosteric repulsion. Encapsulation study performed on these niosomes also yielded a good result, given approximately 80 and 87% encapsulation of DNR and Coumarin-6, respectively.

### *In vitro* drug release

**Figure 1.**
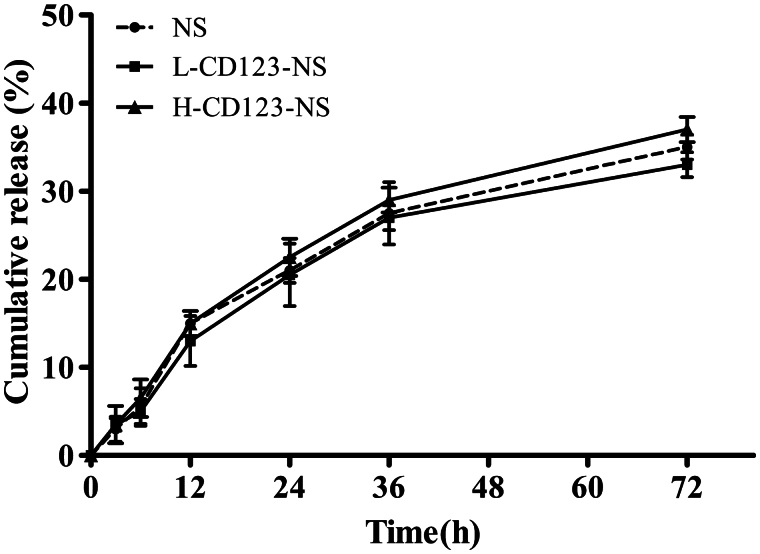
*In vitro* release profiles of DNR from different niosomal formulations in PBS (pH 7.4) at 37 °C. Data represent mean ± SD (*n* = 3).

The *in vitro* release kinetics of DNR from NS, L-CD123-NS and H-CD123-NS were investigated by incubating the above DNR-loaded vesicles in pH 7.4 PBS at 37 °C for up to 72 h. As shown in Figure 1, no significant differences in the DNR release were observed across formulations, suggesting that neither antibody modification nor its density affected the release kinetics of DNR from the prepared niosomes. About 37.5 and 33.8% of DNR was released cumulatively from H-CD123-NS and L-CD123-NS, respectively, within 72 h, whereas 35.6% of DNR was released from NS under the same conditions. There was no significant burst phase across formulations during test period since the drug release was relatively limited for up to 72 h. Additionally, the high stability of that niosomal formulations in which majority of DNR was maintained by 72 h that ensure sufficient drug delivery into AML cells in the form of noisome before the drug release.

### CD123 expression on AML cells

**Figure 2.**
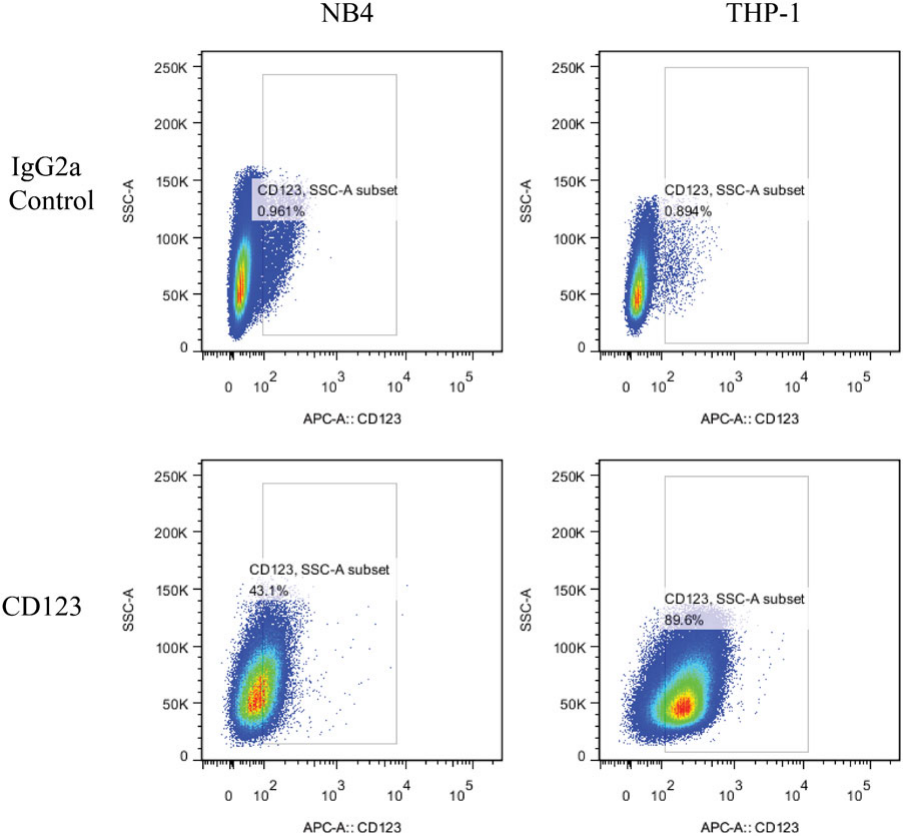
Quantification of the total and surface expression levels of CD123 in NB4 and THP-1 cells, respectively. Numbers indicate percentages of positive cells. IgG2a was set as an isotype control of anti-CD123 antibody.

The CD123 on surface of NB4 and THP-1 cells were quantified through Multi-channel flow cytometry. IgG2a was set as an isotype control of anti-CD123 antibody. As shown in Figure 2, both THP-1 and NB4 cells showed varying levels of CD123 expression, THP-1 (89.6%) and NB4 (43.1%) cells (*p* < .01). These cell lines were then utilized to study the CD123-specific targeting of niosomes.

### CD123-specific targeting and uptake of CD123-NS

**Figure 3.**
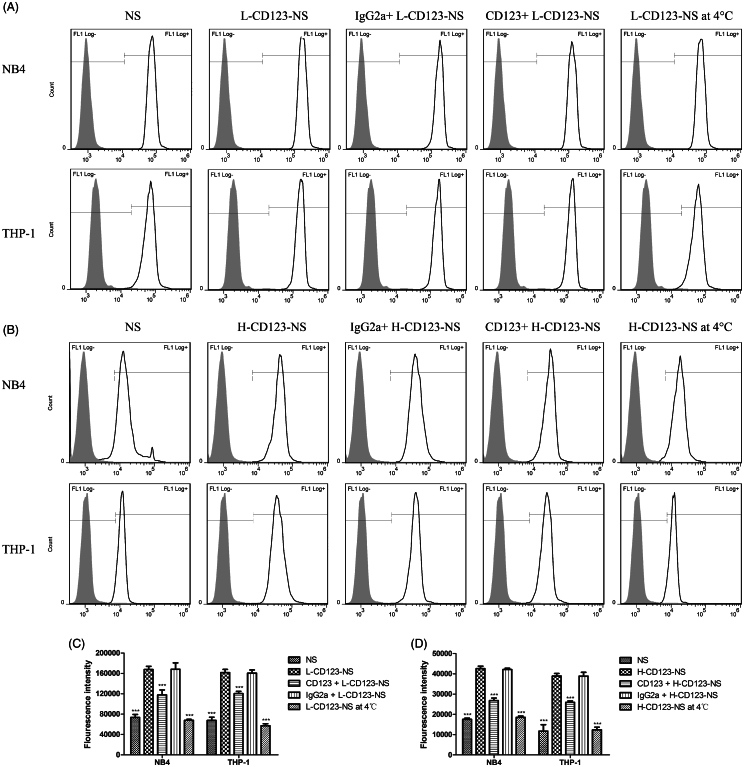
Quantitative determination of the cellular uptake of each Coumarin-6-loaded NS group by NB4 and THP-1 cells *in vitro*. (A) The uptake of L-CD123-NS (final Coumarin-6 concentration of each sample was 40 ng/ml) in NB4 and THP-1 cells. (B) The uptake of H-CD123-NS (final Coumarin-6 concentration of each sample was 10 ng/ml) in NB4 and THP-1 cells. (C) Summary of L-CD123-NS cellular association in NB4 and THP-1 cells. (D) Summary of H-CD123-NS cellular association in NB4 and THP-1 cells. ∗∗∗Indicate *p* < .001 versus the CD123-NS group, each bar represents mean ± SD (*n* = 3). CD123 + and IgG2a + mean the prior presence of free anti-CD123 antibody or IgG2a isotype control antibody for competition experiments.

To study the uptake efficiency of NS formulations, Coumarin-6 was used as the fluorescent probe and encapsulated in NS, L-CD123-NS and H-CD123-NS. Flow cytometry analysis showed that the fluorescence intensities of CD123-NS in both NB4 and THP-1 cells were significantly higher than that of NS, and the uptake efficiency of the CD123-NS increased with the antibody densities (Figure 3). Specifically, the mean fluorescence intensities of L-CD123-NS and H-CD123-NS in NB4 cells were 2.27-fold and 2.43-fold higher than that of NS. Similarly, the mean fluorescence intensities of L-CD123-NS and H-CD123-NS taken up by THP-1 cells were 2.39-fold and 3.31-fold higher than that of NS. Our data showed cellular uptake efficiency of CD123-NS and NS by NB4 cells were higher than those by THP-1 cells, which could be ascribed to inherent uptake differences between the two cell lines. The presence of anti-CD123 antibody has remarkable effect on the cell uptake of niosomes, especially for THP-1 cells, indicating an enhanced uptake via the CD123-dependent endocytosis *in vitro*.

Contrary to a pronounced intracellular accumulation of CD123-NS at 37 °C, the cellular uptake efficiency of Coumarin-6 in two types of CD123-NS was significantly reduced in both NB4 and THP-1 cells when temperature was reduced to 4 °C (Figure 3), indicating the endocytosis of niosomes is energy-driven. Approximately 59.5 and 64.6% reduction in cellular uptake of L-CD123-NS by NB4 and THP-1 cells were observed at 4 °C, respectively (*p* < .001), and the uptake efficiency of H-CD123-NS in NB4 and THP-1 cells at 4 °C reduced by 56.4 and 68.0%, compared to that at 37 °C respectively (*p* < .001). As reported previously (Dinauer et al., 2005; Laginha et al., 2005), the mean fluorescence intensity of Coumarin-6 associated with two cell types at 37 °C presented a combination of binding and receptor-mediated internalization of CD123-NS, while at 4 °C only showed the binding of antibody-targeted niosomes to cell surface antigens. Based on these findings, we conclude that this temperature dependency cellular uptake of CD123-NS by NB4 and THP-1 cells should be ascribed to receptor-mediated endocytosis.

### Competition experiments

**Figure 4.**
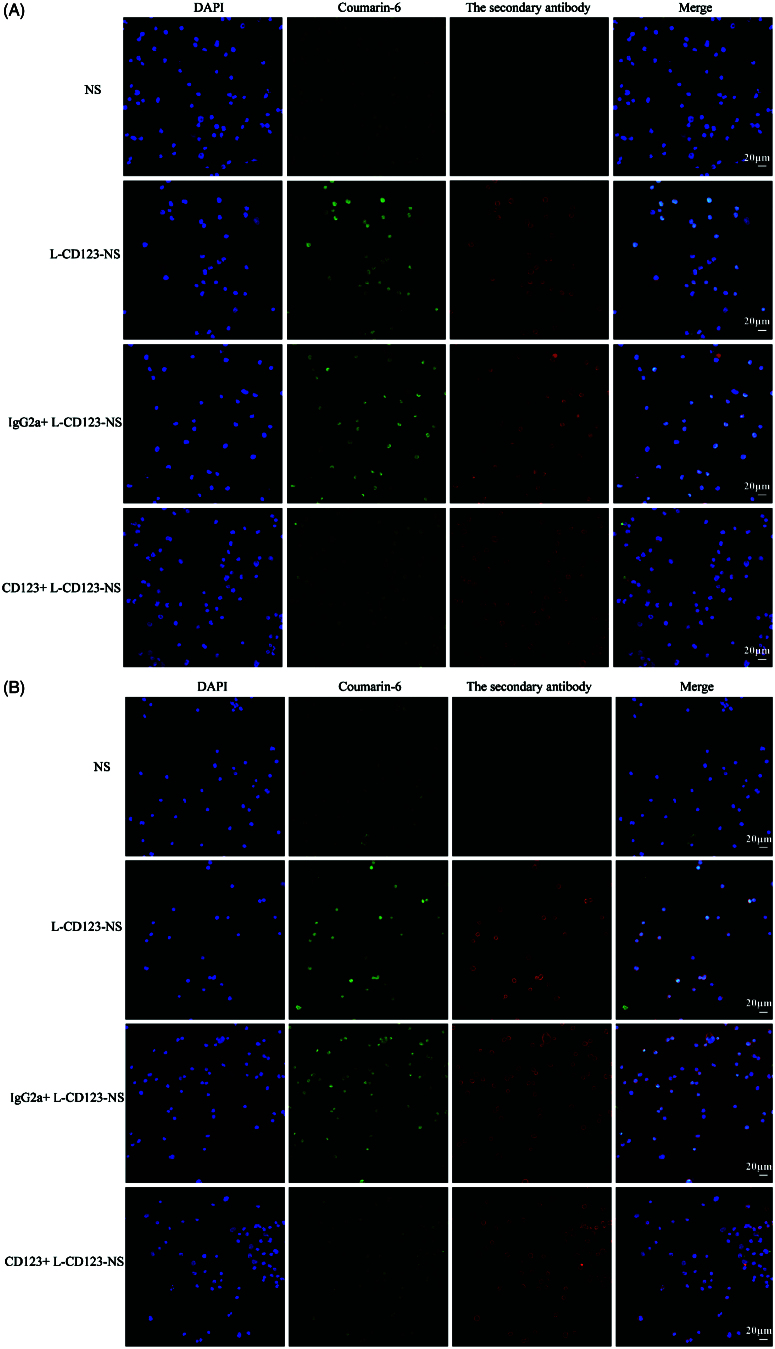
Cellular distribution of Coumarin-6-loaded NS, L-CD123-NS, IgG2a + L-CD123-NS and CD123 + L-CD123-NS in (A) NB4 and (B) THP-1 cells at 37 °C. CD123 + and IgG2a + mean the prior presence of free anti-CD123 antibody or IgG2a isotype control antibody for competition experiments.

To confirm the findings of CD123-specific uptake of the targeted niosomes, competition experiments were carried out in NB4 and THP-1 cells with preincubation of free IgG2a isotype control antibody or anti-CD123 antibody. Theoretically, competition for binding to available CD123 active sites on the cell surface should take place between free and niosomes-bound anti-CD123 antibody. CLSM imaging showed that pretreatment with free anti-CD123 antibody significantly reduced uptake of L-CD123-NS in NB4 and THP-1 cells, whereas preincubation with IgG2a isotype control antibody did not change the uptake efficiency of L-CD123-NS in NB4 and THP-1 cells (Figure 4(A,B)), indicating a competition between anti-CD123 antibody on the CD123-NS and free anti-CD123 antibody for the CD123 antigens present on the cell surface. The quantitative results further revealed that the presence of competitive inhibitors suppressed approximately 29.7 and 36.9% cellular uptake of L-CD123-NS and H-CD123-NS in NB4 cells, respectively (Figure 3(C,D)). A similar trend in THP-1 cells has also been observed that about 25.5% of L-CD123-NS and 33.3% of H-CD123-NS uptaken by THP-1 cells were inhibited, respectively. Thus, the internalization of CD123-NS in AML cells was likely via a CD123-dependent endocytosis pathway.

### Cytotoxicity of DNR-loaded niosomes

Prior studies in our group have indicated a therapeutic benefit of peptide modified active DNR delivery system in the treatment of AML (Liu et al., 2014). In this study, the effect of anti-CD123 antibody decoration on the cytotoxicity of DNR-loaded niosomes was evaluated in both NB4 and THP-1 cells after 24 h incubation. Both two types of DNR-loaded CD123-NS exhibited markedly elevated inhibitory effect on the proliferation of NB4 and THP-1 cells in all tested concentrations. As shown in Table 1, the IC_50_ of DNR-NS was decreased nearly 1.36-fold and 1.45-fold comparing to free DNR in NB4 and THP-1 cells, respectively. Remarkably, H-CD123-NS induced a 2.45-fold and 3.22-fold greater DNR cytotoxicity than NS in NB4 and THP-1 cell, respectively, while L-CD123-NS induced a 2.17-fold and 2.36-fold greater DNR cytotoxicity, respectively. The results above were consistent with the uptake study, indicating that the CD123 targeting greatly improved the delivery efficiency of DNR to the targeted cells.

**Table 1.** IC_50_ of different treatment groups to DNR in NB4 and THP-1 cells.

IC_50_ (μM)	DNR	DNR-NS	DNR-L-CD123-NS	DNR-H-CD123-NS
NB4	5.38 ± 0.05***	3.97 ± 0.13***	1.83 ± 0.08*	1.62 ± 0.04
THP-1	5.00 ± 0.11***	3.44 ± 0.09***	1.46 ± 0.10***	1.07 ± 0.06

Data represent mean ± SD (*n* = 3). *p < .05 and ^***^p < .001 versus the DNR-H-CD123-NS group.

### *In vivo* survival experiment

**Figure 5.**
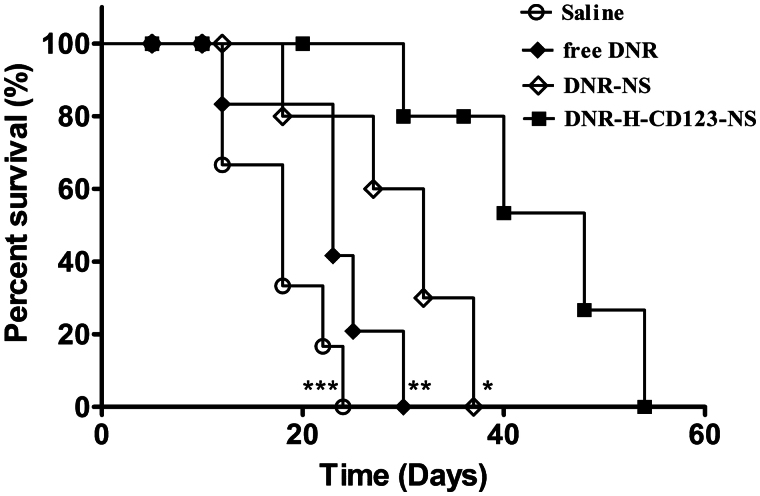
Therapeutic activity of DNR-H-CD123- NS in THP-1 bearing NOD/SCID mice (*n* = 8). Animals treated intravenously with DNR-H-CD123- NS (3 mg/kg DNR) survived significantly longer than mice treated with saline, free DNR and DNR-NS. ***p < .001, **p < .01 and *p < .05 versus the DNR-H-CD123- NS group, respectively (long-rank test).

To evaluate the anti-tumor efficacy of niosomal formulations *in vivo*, a survival study was performed in THP-1-bearing NOD/SCID mice. As shown in Figure 5, the mice treated with DNR-H-CD123-NS survived significantly longer than those treated with saline (*p* = .0007), free DNR (*p* = .0045) or DNR-NS (*p* = .0340). The median survival times for the four groups (saline, free DNR, DNR-NS and DNR-H-CD123- NS) were 18, 23, 32 and 48 days, respectively. The improved therapeutic performance of niosomal formulations *in vivo* may be attributed to selectively DNR delivery through CD123-mediated endocytosis that allow more drug molecules to enter the AML cells. This is the first time anti-CD123 antibody-modified niosomes have been utilized in AML mouse models to show the admirable therapeutic effect over conventional chemotherapy. We suggest that targeted drug delivery for blood tumors such as AML may be profitable for easy access to tumor cells, without overcoming the multiple physical barriers that are inevitable for solid tumors.

## Conclusion

In this study, CD123-NS, a novel niosomal drug delivery system modified with anti-CD123 antibodies was developed for targeted drug delivery to AML cells. Niosome-antibody conjugate was successfully constructed by post-insertion method and the biological activity of anti-CD123 antibody on the niosomes was well preserved. CD123-NS exhibited an elevated cellular uptake efficiency and enhanced cytotoxicity on CD123 over-expressed NB4 and THP-1 cells compared to the NS. Moreover, *in vivo* studies further demonstrated the superior targeting ability and therapeutic effect of DNR-loaded CD123-NS. Therefore, anti-CD123 antibody-conjugated niosomes (CD123-NS) represent a promising targeted therapy against AML.
